# Amplicon-Based Analysis of the Fungal Diversity across Four Kenyan Soda Lakes

**DOI:** 10.1155/2022/9182034

**Published:** 2022-05-05

**Authors:** Romano Mwirichia

**Affiliations:** Department of Biological Sciences, University of Embu, P.O Box 6-60100, Embu, Kenya

## Abstract

Microorganisms have been able to colonize and thrive in extreme environments characterized by low/high pH, temperature, salt, or pressure. Examples of extreme environments are soda lakes and soda deserts. The objective of this study was to explore the fungal diversity across soda lakes Magadi, Elmenteita, Sonachi, and Bogoria in Kenya. A new set of PCR primers was designed to amplify a fragment long enough for the 454-pyrosequencing technology. Analysis of the amplicons generated showed that the new primers amplified for diverse fungal groups. A total of 153,634 quality-filtered, nonchimeric sequences derived from the 18S region of the rRNA region were used for community diversity analysis. The sequence reads were clustered into 502 OTUs at 97% similarity cut-off using BLASTn analysis of which 432 were affiliated to known fungal phylotypes and the rest to other eukaryotes. Fungal OTUs were distributed across 107 genera affiliated to the phyla Ascomycota, Basidiomycota, Glomeromycota, and and other unclassified groups refred to as Incertae sedis. The phylum *Ascomycota* was the most abundant in terms of OTUs. Overall, fifteen genera (*Chaetomium*, *Monodictys*, *Arthrinium*, *Cladosporium*, *Fusarium*, *Myrothecium*, *Phyllosticta*, *Coniochaeta*, *Diatrype*, *Sarocladium*, *Sclerotinia*, *Aspergillus*, *Preussia,* and *Eutypa*) accounted for 65.3% of all the reads. The genus *Cladosporium* was detected across all the samples at varying percentages with the highest being water from Lake Bogoria (51.4%). Good's coverage estimator values ranged between 97 and 100%, an indication that the dominant phylotypes were represented in the data. These results provide useful insights that can guide cultivation-dependent studies to understand the physiology and biochemistry of the as-yet uncultured taxa.

## 1. Introduction

Microorganisms have been able to not only colonize but also thrive under unique or extreme environmental conditions, which characterized by low/high pH, temperature, salt, or pressure. Examples of extreme environments are soda lakes, which are characterized by high alkalinity (with pH values ranging between 9 and 12 to the point where Na^+^ concentrations can reach saturation). Their surface area fluctuates due to extensive evaporation attributed to the intense evaporation and low levels of precipitation experienced where they are located. Despite the extreme physicochemical conditions in the soda lake ecosystems, a high level of species diversity has been reported [1–4]. These habitats may exhibit higher productivity than freshwater bodies [5].

Whereas most of the studies on extremophiles have focused on prokaryotes, there are reports of alkaliphilic and alkalitolerant fungi isolated from soda lakes and soda soils in different parts of the world [6–9]. Different species of black yeast have been isolated from hypersaline waters of solar saltans [10, 11]. Different genera, including *Cladosporium, Aspergillus, Penicillium, Alternaria,* and *Acremonium*, have been reported to exist as either moderately or weakly alkali tolerant species in saline environments [8]. Isolates affiliated to *Chaetomium aureum*, *C. flavigenum*, *Emericella nidulans*, and *Eurotium amstelodami* have previously been isolated from the Dead Sea [12]. Orwa et al. [6] described isolates distributed over 18 fungal genera, namely, *Aspergillus*, *Penicillium*, *Acremonium*, *Phoma*, *Cladosporium*, *Septoriella*, *Talaromyces*, *Zasmidium*, *Chaetomium*, *Aniptodera*, *Pyrenochaeta*, *Septoria*, *Juncaceicola*, *Paradendryphiella*, *Sarocladium*, *Phaeosphaeria*, *Juncaceicola*, and *Biatriospora* from Lake Magadi in Kenya. Other reports include *Chaetomium globosum* from the Dead Sea and saline habitats of Wadi El Natrun [13] and Sarocladium kiliense from Lake Sonachi in Kenya [14].

High-throughput sequencing allows rapid estimation and identification of microorganisms without cultivation [15]. Using this approach, a high prokaryotic and eukaryotic diversity has been reported from several alkaline lakes such as Magadi in Kenya [16, 17], Ethiopian soda lakes [1], Central European hypersaline lakes [18], and sediments from the Tibetan Plateau [19]. Therefore, a sequence-based approach has made it easier to understand diversity and structure of microbial communities in diverse environments [20, 21]. Most of the high-throughput sequencing technologies used in diversity studies have an amplification step. The earliest PCR primers to gain wide acceptance in fungal studies were designed for the nuclear ribosomal internal transcribed spacer (ITS) region and were described by White et al. [22] and thereafter modified by several researchers [23–25]. However, the ITS region does not suffice to resolve the species level in some groups of fungi [15, 23, 26–28].

Besides the ITS region, the small (18S/SSU) and large (28S/LSU) subunits of the rRNA operon have been targeted for amplification in fungal diversity studies [15, 29]. Several group-specific 18S rRNA gene primers have been developed [30–32]. However, coverage and phylogenetic resolution to lower taxonomic levels are always a challenge, especially when dealing with less explored habitats. In this study, we designed a new set of primers targeting the 18S rRNA gene for high-throughput sequencing and tested them using various samples collected from different soda lakes in Kenya. The main objective was to explore whether fungal diversity varies between the lakes Magadi, Elmenteita, Sonachi, and Bogoria and across each lake due to differences in physicochemical parameters. The study provides new insights into the spatial diversity across various soda lakes and with varying physicochemical parameters.

## 2. Materials and Methods

### 2.1. Description of Study Sites and Sampling Design

Study sites chosen for the study were the hypersaline lake Magadi (2^o^00′S and 36^o^13′E) at an elevation of 600 m above sea level. It lies in a naturally formed closed lake basin and has an annual rainfall of approximately 500 mm [33]. The lake covers an area of 90 km^2^, and evaporation is intense during the dry season. Lake Elmenteita (0°27′S, 36°15′E) is a moderately saline lake located 1776 m above sea level. It has no direct outlet. The lake is approximately 20 km^2^, but the total surface area changes with seasons. The lake often floods during heavy rains. Lake Bogoria (0° 20′N and 36° 15′E) lies at an altitude of 975 m. It has a low rainfall of 708 mm, and there are several geysers around the lake. The alkaline, saline crater lake Sonachi, lies in a closed basin on the Eastern Rift valley (0° 49′S, 36° 16′E).

### 2.2. Sample Collection and Nucleic Acid Extraction

Wet sediment, water samples, microbial mats, dry sediments, and grassland soil were collected from lakes Bogoria, Elmenteita, Sonachi, and Magadi as described [6]. 1 g of each soil or sediment sample was weighed into a sterile Eppendorf tube. For the water samples, 500 ml was filtered through a 0.22-*μ*m filter, cut into small pieces with a sterile scalpel, and transferred to a sterile 2-ml tube. Total DNA was extracted using the phenol: chloroform protocol [34]. However, proteinase K was substituted with 6 M guanidine isothiocyanate (GITC) for protein denaturation. Our experience is that extraction of high molecular weight DNA from the soda lake samples using kits is problematic due to high salt content in the samples.

### 2.3. Primer Design and PCR Amplification

A nonredundant set total of 270 fungal sequences representing the 18S rRNA marker gene was downloaded from the SILVA database [35, 36] and aligned using ClustalW v2.1 [37] available from the EMBL-EBI. A consensus sequence was generated using Jalview v2.10 [38] and used to design the reverse and forward primer using GeneFischer2 (https://bibiserv.cebitec.uni-bielefeld.de/genefisher2) described by Giegerich et al. [39]. Both primers were tested in Probe Match (https://rdp.cme.msu.edu/probematch/search.jsp) [40]. The new primer pair amplifies a fragment of 712 bases covering the V3, V4, V5, and V6 regions [41] of the 18S rRNA gene as well as partial V2 and V7 regions. The designed primers are designated as Fung_576f (5′-GCTCGTAGTTGAACCTTTGG-3′) and Fung_975r (5′-TCTGGACCTGGTGAGTTTC-3′). Thereafter, the primers were modified for pyrosequencing by attaching an adaptor sequence, a key, and a unique 12 nucleotide MID for multiplexing purposes. Each PCR (50 *μ*L) contained forward and reverse primers (10 *μ*M each), dNTP's (10 mM each) Phusion GC buffer (Finnzymes), Phusion high-fidelity polymerase (0.5 U/*μ*L^−1^), and 25 ng of template DNA. Cycling conditions were as follows: initial denaturation at 98°C for 3 minutes followed by 25 cycles of denaturation at 94°C for 30 sec, annealing for 30 sec at 58°C, extension at 72°C for 90 sec, and a final extension step of 72°C for 5 min. Amplification was confirmed by separating 2 *μ*l of the PCR product on a 1% TAE agarose gel (40 mM Tris base, 20 mM glacial acetic acid, 1 mM EDTA, and 1.5% (w/v) agarose) run for 1 h at 100 V. Later, three independent PCR products per sample were pooled in equal amounts, separated on a gel, and extracted using the PeqGOLD gel extraction kit (PeqLab Biotechnologie GmbH, Erlangen, Germany). PCR products were quantified using a Nanodrop (PEQLAB Biotechnologie GmbH, Erlangen, Germany) and a Qubit fluorometer (Invitrogen GmbH, Karlsruhe, Germany) as recommended by the manufacturers. Sequencing of the PCR derived amplicons was performed on a Roche GS-FLX 454 pyrosequencer and Titanium chemistry (Roche, Mannheim, Germany) at Göttingen Genomics Laboratory, Georg August University Göttingen, Germany. The raw sequence reads have been deposited into the SRA under the accession SRP019052.

### 2.4. Sequence Analysis

Sequence reads were denoised and evaluated for potential chimeric sequences using UCHIME within the USEARCH package v.11.0 [42]. OTU picking was done from the quality filtered, denoised, and nonchimeric sequences using a sequence identity cutoff of 97%. Representative OTUs were picked using vsearch v2.14 [43]. Taxonomy was assigned to the representative sequences from each cluster by BLAST searches against the SILVA database of ARB version 132 [35, 36].

### 2.5. Statistical Analysis

Rarefaction analysis using the script *alpha_rarefaction.py* in QIIME [44] was done to assess whether the sampling effort was representative of the microbial diversity in the samples. OTUs were assigned to ecological guilds using the annotation tool FUNGuild [45]. A heatmap to display the most abundant OTUs was created using the ampvis2 R package [46]. Diversity estimates (Good's coverage, Chao1, and Shannon) were generated using the *alpha_diversity.py* script in QIIME [44]. The package “*indicspecies*” [47] in R was used to find out whether there were genera that were significantly associated with different sample types. Nonmetric multidimensional scaling (NMDS) of fungal communities was conducted in R using the vegan package [48] based on unweighted UniFrac [49] distance matrices.

## 3. Results

### 3.1. Evaluation of the New Primer Set

The newly designed primers amplified for eukaryotic groups only, and no bacterial sequences were detected in this study. In each sample, the success rate for amplifying for fungal groups was above 90%, which was considered good for environmental DNA. The amplicons could also be assigned to taxonomy with high confidence owing to the sequence length generated using the 454 technology. In addition, the samples used ranged from dry sediments to microbial mats, and therefore, good quality DNA is key to amplification.

### 3.2. Sequence Data

The clean data from 32 samples comprised 153,634 quality-filtered, denoised, and nonchimeric sequences with no singletons. Of these, 152,834 were fungal amplicons, which represent a primer success rate of 99.48%. These fungal sequences were clustered into 502 OTUs at 97% similarity of which 417 were affiliated to known fungal phyla. The remaining OTUs represented Bacillariophyta (25), Streptophyta (1 OTU), and other unclassified eukaryotic groups termed *Incertae Sedis* with 6 OTUs. Fungal OTUs per sample ranged from 13 in the Bogoria wet sediments (sample BWS10) to 68 in the dry sediments from Lake Sonachi (sample BDS10) as shown in Table 1.

### 3.3. Diversity at the Phylum Level

**Figure 1:**
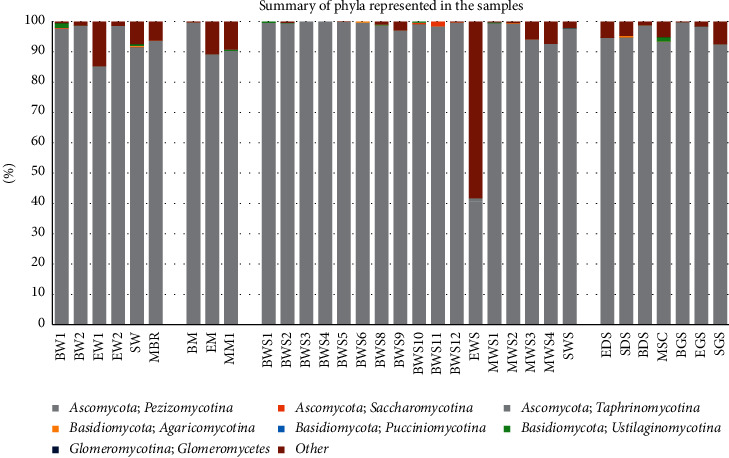
Distribution of OTUs at the class level across the 33 samples analyzed in this study. BW, EW, and SW denote water samples from Bogoria, Elmenteita, and Sonachi, respectively; BWS, EWS, MWS, and SWS denote wet sediment samples from Bogoria, Elmenteita, Magadi, and Sonachi, respectively; EDS and SDS denote dry sediments from Elmenteita and Sonachi, respectively; BM and EM denote microbial mats from Bogoria and Elmenteita, respectively, while MBR and MSC denote brine and salt crust from Magadi; BGS, EGS, and SGS denote grassland soils from Bogoria, Elmenteita, and Sonachi, respectively.

The 417 fungal OTUs with assigned taxonomy were distributed across 113 fungal genera affiliated to the phyla Ascomycota, Basidiomycota, Glomeromycota, and a smaller percentage to other nonfungal eukaryotic groups (Figure 1). The phylum Ascomycota was the most dominant phylum, and its orders *Capnodiales*, *Pleosporales*, *Hypocreales*, *Myrmecridiales*, *Sordariales*, and *Xylariales* were the most abundant. In this phylum, the order *Pleosporales* was the most diverse with 13 genera followed by the orders *Capnodiales* (9 genera), *Hypocreales* (11 genera), and *Xylariales* (11 genera). However, we noted that in Elmenteita wet sediments (sample EWS), the nonfungal phylum Bacillariophyta accounted for 45% of the OTUs (Figure 1).

**Figure 2:**
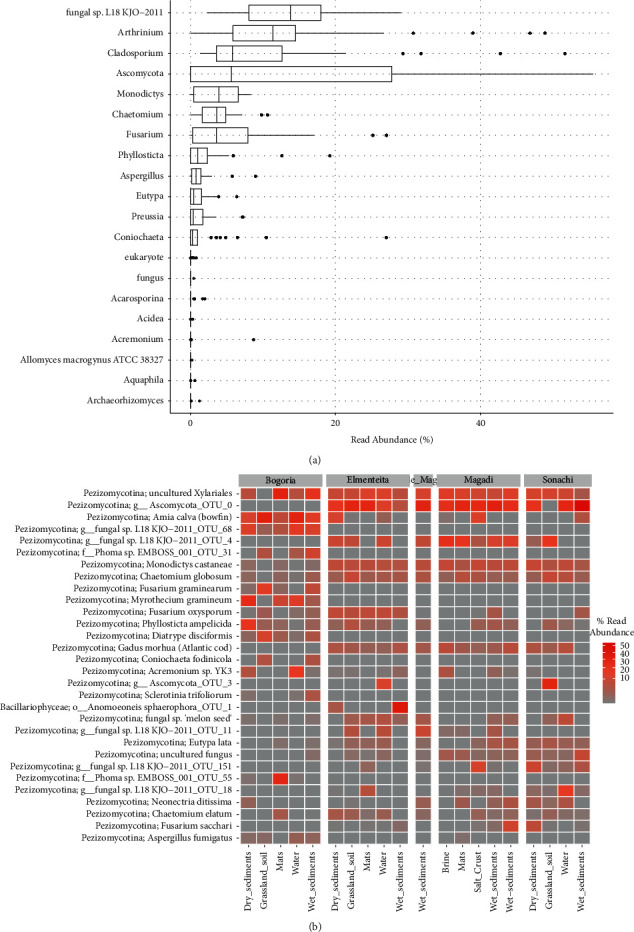
(a) Percentage read abundance of the top 20 species across the samples. (b) Heatmap showing the % abundance of the top 30 phylotypes.

Sequences affiliated to Basidiomycota were detected in a few of the samples and were distributed in the orders *Agaricales*, *Boletales*, *Polyporales*, *Cystofilobasidiales*, *Filobasidiales*, *Sporidiobolales*, and *Malasseziales*. The phylum *Glomeromycota* was represented by a single species—*Diversispora eburnean*—that was detected in the Elmenteita grassland soil. Overall, we identified fifteen (15) ascomycete genera that constituted 65.3% of all the reads and these were *Arthrinium*, *Cladosporium*, *Fusarium*, *Chaetomium*, *Monodictys*, *Myrothecium*, *Phyllosticta*, *Coniochaeta*, *Diatrype*, *Sarocladium*, *Sclerotinia*, *Aspergillus*, *Preussia*, and *Eutypa* (Figure 2(a)). The genus *Cladosporium* was detected across all the samples at varying percentages with the highest being water from Lake Bogoria (51.4%). However, at the species level, the most abundant reads were affiliated to uncultured phylotypes in the class Pezizomycotina (Figure 2(b)).

### 3.4. Diversity across the Different Samples

**Figure 3:**
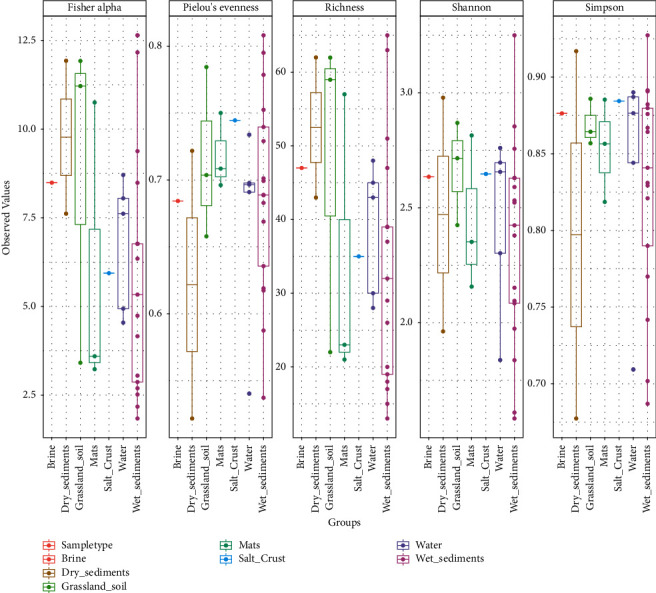
Alpha diversity indices across the samples based on richness, Simpson, Shannon, evenness, and Fisher.

We evaluated and compared the diversity across the 32 samples. We used different metrics (Richness, Simpson, Shannon, Evenness, Fisher, and Good's coverage) to evaluate the alpha diversity across the samples. Good's coverage estimator values were between 97 and 100% (Table 1). This is an indication that the dominant phylotypes were represented in the data. This was even more evident when the samples were clustered by sample type (*P* = 0.05). The lowest diversity was found in the brine samples and the highest in the sediment samples (Figure 3).

### 3.5. Differences in Fungal Diversity across the Lakes

**Figure 4:**
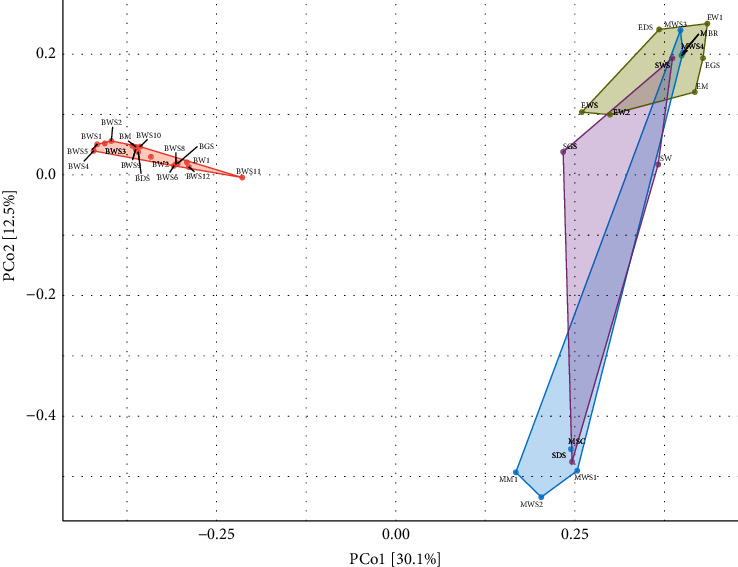
Bray–Curtis dissimilarity analysis showing the clustering of the samples.

Bray–Curtis dissimilarity analysis (Figure 4) demonstrated that the samples were separated into 3 clusters. The samples from Lake Bogoria formed a distinct cluster. This could be due to differences in OTU composition. For example, no OTUs related to the genus *Cladosporium* were detected in samples from Lake Bogoria, whereas the genera *Myrothecium, Sclerotinia, Lasiodiplodia*, and *Peziza* were only recovered from Lake Bogoria samples.

Indicator species analysis identified 5 OTUs affiliated to the genus *Ochroconis* (*P*=0.01), *Aspergillus* (*P*=0.01), *Cladosporium* (*P*=0.01), and *Sarocladium* (*P*=0.001) as key species in the brine samples only. Using FUNGuild, 63.6% of the fungal OTUs were classified according to their trophic mode as either saprotroph (32%), pathotrophs (19%), or symbiotrophs (9%). The rest were classified as saprotroph-symbiotroph (2%), pathotroph-symbiotroph (4%), pathotroph-saprotroph (7%), and pathotroph-saprotroph-symbiotroph (28%).

## 4. Discussion

The overall diversity and significance of fungal communities in the soda lakes have not been understood owing to the limited data available as compared to bacteria. The Kenyan soda lakes are situated in geographically remote areas that experience intense solar radiation; evaporation rates exceed precipitation rates; hence, there is a concentration of salts, which contributes to the elevated salinity levels. This may be one of the reasons why they are not well explored. The diversity reported so far has been based on culture dependent studies [6, 50, 51] or using molecular approaches [16, 17]. Amplicon sequencing provides a better and more detailed understanding of the fungal diversity than does cultivation in these unique habitats. Out of the 432 fungal OTUS, 389 were identified to the genus level, while 320 were tentatively identified to the species level. Additionally, 3% of the fungi detected could not be classified and may represent novel autochthonous soda lake fungal phylotypes. In microbial diversity studies, reports show that a major percentage of the observed species are tagged as uncultured [52–55]. This necessitates more isolation or genome sequencing efforts to understand the physiology and metabolism of these novel groups.

In this study, the phylum *Ascomycota* accounted for more than 80% of the reads across the sample, with the most abundant classes being *Dothideomycetes*, followed by *Sordariomycetes, Leotiomycetes*, *Eurotiomycetes,* and *Pezizomycetes*. Sharma et al. [56] reported that 98% of the isolates recovered from Lonar lake belonged to *Ascomycota*, subphylum *Pezizomycotina*. Ascomycetes have also been reported to be dominant in marine sediments of Kongsfjorden, Svalbard [57], constituting 54.8% of the OTUs. In marine sediments of the Arabian Sea, *Ascomycota* were reported to be the most abundant phylum at 83% and the rest (17%) were *Zygomycota* [58]. However, *Basidiomycota* has been reported to be dominant in other hyper-saline environments [59, 60]. The genus *Cladosporium* was detected across all the samples with the highest relative abundance being in water from Lake Bogoria (51.4%). However, some of the phylotypes (*Cladosporium sphaerospermum*, *Fusarium* sp., and *Penicillium* sp.) have also been observed in marine sediments [58, 61]. Chaetomium globosum has been isolated from the Dead Sea as well as saline habitats of Wadi El-Natrun [13], while isolates with close similarity to Sarocladium kiliense were recovered Lake Sonachi sediments [14]. Salinity and pH have an impact on fungal growth and spore formation, which in turn may affect the overall diversity [5]. Production of extremolytes and extremozymes, and accumulation of K ions and compatible organic solutes in the cells are ways of coping with osmotic stress [62–64]. Unique features such as the thick mycelium observed in Phoma herbarum are important in stress tolerance, while pigments such as those produced by Zasmidium cellare, Aspergillus keveii, and Cladosporium velox enable them to thrive in the harsh environments [6, 65]. Previous culture-dependent studies [6] of Lake Magadi recovered isolates distributed across several fungal genera, namely, *Aspergillus*, *Penicillium*, *Acremonium*, *Phoma*, *Cladosporium*, *Septoriella*, *Talaromyces*, *Zasmidium*, *Chaetomium*, *Aniptodera*, *Pyrenochaeta*, *Septoria*, *Juncaceicola*, *Paradendryphiella*, *Sarocladium*, *Phaeosphaeria*, *Juncaceicola,* and *Biatriospora*. These isolates grew better when lake water was used in media preparation as compared to synthetic mineral medium. This may be an indication that they are adapted to the haloalkaline environment.

## 5. Conclusion

A key ecological question is whether the observed fungal groups originate from the terrestrial environment via runoff or are actual residents exclusive to the soda lake habitats. It is possible that runoff from the surrounding soil introduces spores into the lakes; any such species may over time have adapted to the haloalkaline environment. In summary, the diversity and function of most fungal taxa in the soda lake ecosystem remain poorly understood. Therefore, a combination of traditional culture-based method and metatranscriptomics may help to answer important ecological questions.

## Figures and Tables

**Table 1:** Summary description of the 33 samples investigated and their diversity indices.

Sample ID	Lake	Sample type	pH	Temp. (°C)	Total no. of sequences	Fungal sequences	% of fungal sequences	Total no. of OTUs	Fungal OTUs	Chao1	Good's coverage
BDS	Bogoria	Wet_sediments	7.6	34	2,340	2,320	99.15	47	47	41.75	0.99
BGS	Bogoria	Grassland_soil	7.6	24	661	658	99.55	26	22	24.00	1.00
BMM	Bogoria	Mats	7.6	72	1,073	1,073	100.00	21	21	22.60	0.99
BW1	Bogoria	Water	7.6	88	3,401	3,394	99.79	39	30	34.00	0.98
BW2	Bogoria	Water	10.1	28	1,467	1,446	98.57	34	28	28.00	0.99
BWS1	Bogoria	Wet_sediments	7.6	24.3	736	735	99.86	20	17	17.00	1.00
BWS2	Bogoria	Wet_sediments	7.6	27	1,103	1,093	99.09	21	18	18.00	1.00
BWS3	Bogoria	Wet_sediments	7.6	30.6	747	747	100.00	15	15	15.00	1.00
BWS4	Bogoria	Wet_sediments	7.6	42.6	1,612	1,601	99.32	34	32	30.00	0.99
BWS5	Bogoria	Wet_sediments	7.6	55.8	764	756	98.95	23	19	20.00	1.00
BWS6	Bogoria	Wet_sediments	7.6	68.1	1,032	1,029	99.71	23	20	19.25	1.00
BWS8	Bogoria	Wet_sediments	7.6	76.1	2,637	2,592	98.29	41	37	39.50	0.99
BWS9	Bogoria	Wet_sediments	7.6	44.6	2,067	2,052	99.27	30	28	36.00	0.98
BWS10	Bogoria	Wet_sediments	7.6	35.8	596	595	99.83	14	13	13.00	1.00
BWS12	Bogoria	Wet_sediments	10.1	72	3369	3,338	99.08	47	39	36.00	0.99
EDS	Elmenteita	Dry_sediments	9.9	24	18,264	18,108	99.15	78	60	52.43	0.98
EGS	Elmenteita	Grassland_soil	9.9	24	8,177	8,148	99.65	62	59	64.00	0.97
EM	Elmenteita	Mats	9.9	65	6,745	6,732	99.81	65	58	48.43	0.98
EW1	Elmenteita	Water	9.9	65	12,992	12,881	99.15	52	46	38.75	0.99
EW2	Elmenteita	Water	8.7	24	6,776	6,746	99.56	58	45	45.25	0.98
EWS	Elmenteita	Wet_sediments	9.9	22.7	15,612	15,598	99.91	68	40	46.00	0.98
MBR	Magadi	Brine	10.3	37.4	9,988	9,928	99.40	51	47	37.88	0.99
MM1	Magadi	Mats	9.4	44.9	522	520	99.62	27	23	26.17	1.00
MSC	Magadi	Salt_Crust	10.3	47.2	1,181	1,174	99.41	40	34	39.00	0.99
MWS1	Magadi	Wet_sediments	9.4	44.9	1,062	1,052	99.06	40	39	38.60	0.99
MWS2	Magadi	Wet_sediments	9.4	44.9	585	582	99.49	28	26	27.25	1.00
MWS3	Magadi	Wet_sediments	8.5	38	12,121	12,121	100.00	39	39	33.50	0.99
MWS4	Magadi	Wet_sediments	9.4	85	11,499	11,481	99.84	73	64	62.00	0.97
SDS	Sonachi	Wet_sediments	9.9	26.9	1,561	1,538	98.53	81	68	66.59	0.97
SGS	Sonachi	Grassland_soil	9.9	26.9	7,608	7,571	99.51	72	63	56.11	0.97
SW	Sonachi	Water	9.9	26.9	5,400	5,370	99.44	59	48	39.14	0.99
SWS	Sonachi	Dry_sediments	9.9	26.9	9,936	9,855	99.18	52	43	39.50	0.98

The samples are sorted by study site. BW, EW, and SW denote water samples from Bogoria, Elmenteita, and Sonachi, respectively; BWS, EWS, MWS, and SWS denote wet sediment samples from Bogoria, Elmenteita, Magadi, and Sonachi, respectively; EDS and SDS denote dry sediments from Elmenteita and Sonachi, respectively; BM and EM denote microbial mats from Bogoria and Elmenteita, respectively, while MBR and MSC denote brine and salt crust from Magadi; BGS, EGS, and SGS denote grassland soils from Bogoria, Elmenteita, and Sonachi, respectively.

## Data Availability

The raw sequence reads have been deposited into the SRA under the accession SRP019052.
